# Determination of Sulfites in Dried Fruits by Paper Spray Ionization Tandem Mass Spectrometry

**DOI:** 10.3390/molecules29102192

**Published:** 2024-05-08

**Authors:** Donghoon Lee, Heejin Ro, Seoyoung Hwang, Minkyu Lee, Hyebeen Kim, Jaeyoung Heo, Sangwon Cha

**Affiliations:** Department of Chemistry, Dongguk University, Seoul 04620, Republic of Korea

**Keywords:** sulfite, dried fruit, paper spray ionization, tandem mass spectrometry, hydrophilic interaction liquid chromatography

## Abstract

Sulfite, a widely used food additive, is subject to regulated labeling. The extraction of sulfite as the stable hydroxymethylsulfonate (HMS) form and its quantitative analysis by liquid chromatography-tandem mass spectrometry (LC-MS/MS) has been recognized for their good sensitivity, selectivity, and versatility across various food materials. This study aimed to develop a cost-effective and simpler method for sulfite quantitation, while maintaining the superior sensitivity and selectivity of mass spectrometry (MS). To achieve this, we introduced paper spray ionization (PSI), an ambient desorption ionization technique that could achieve the direct measurement of analytes without employing separation. We also employed a novel internal standard (IS) structurally similar to the analyte, replacing the more expensive isotopically labeled IS. Although the PSI-MS/MS method developed in this study exhibited slightly lower analytical performance compared to the conventional LC-MS/MS, it remained effective for sulfite analysis in dried fruits.

## 1. Introduction

Sulfites, a prevalent class of food additives, serve crucial roles in inhibiting fungal growth and preserving the natural color of foods by preventing pigment oxidation [1]. Nonetheless, the identification of sulfite sensitivity among certain individuals has prompted regulatory bodies, including the U.S. Food and Drug Administration (FDA), to require that foods with sulfite concentrations exceeding specific thresholds (such as 10 mg/kg, measured as sulfur dioxide (SO_2_)) be clearly labeled [2].

The accurate quantitation of sulfites is challenging because sulfites exist in unstable free, reversibly bound, and irreversibly bound forms in a variety of complex food matrices. Until 2022, the U.S. FDA mandated the use of the optimized Monier–Williams (MW) method (AOAC Official Method 990.28) for the regulatory analysis of sulfites. This method involves determining sulfite content by capturing and oxidizing SO_2_ released through HCl reflux to produce H_2_SO_4_, which is then measured by titration or gravimetric determination. While the MW method yields accurate results for most food matrices, it is time-consuming, requires specialized glassware, and exhibits low specificity for certain foods, such as *Allium* vegetables [3]. To address these issues, sulfite analytical methods based on various methodologies such as spectrophotometry [4,5], fluorometric analysis [6,7], electrochemical methods [8,9,10], liquid chromatography (LC) [11,12], and LC-tandem mass spectrometry (LC-MS/MS) [1,13] have been developed.

In 2022, the U.S. FDA adopted the LC-MS/MS method [13] from among various alternatives for analyzing sulfites in food materials for regulatory purposes, recognizing its superior versatility, sensitivity, and selectivity [1,13]. In this method, unstable sulfite is extracted and converted to stable hydroxymethylsulfonate (HMS) using a buffered formaldehyde extraction solvent [1,11,13,14]. This is followed by solid-phase extraction (SPE) cleanup and filtration [13]. In addition, specific modification in sample preparation steps according to the moisture or fat content of the target food is also presented [13]. For MS/MS-based quantitation, isotopically labeled sodium sulfite (Na_2_^34^SO_3_) was introduced as the internal standard (IS) to improve the accuracy [13].

In this study, we aimed to develop a more economical and simple method for the analysis of sulfite in food, while maintaining the advantages of the LC-MS/MS-based method mentioned above. To achieve this, we modified two major components of analysis. First, we introduced paper spray ionization (PSI), a simple ambient desorption ionization method instead of an LC-electrospray ionization (ESI) platform. In PSI, a sample solution is applied to a triangular-shaped paper tip connected to a high voltage (HV) supply and dried. A spray/extraction solvent and HV are then applied to the paper tip to extract, transport, spray, and ionize the analytes [15,16,17]. Due to the inherent porosity and filterability of paper tips, PSI-MS usually requires much less sample cleanup than LC-ESI-MS [18]. Consequently, PSI-MS has been extensively employed for the analysis of food ingredients, additives, or contaminants, with minimal sample preparation [19,20,21,22,23,24]. Second, we employed sodium 2-mercaptoethanesulfonate (MES), structurally similar to HMS, as the internal standard (IS), replacing the more costly isotopically labeled sulfite (Na_2_^34^SO_3_).

Our results demonstrated that the developed PSI-MS method for sulfite quantitation delivered analytical performances in terms of accuracy, and precision that are comparable to those of the LC-MS/MS method. Despite PSI-MS having a higher limit of quantitation (LOQ) compared to LC-MS/MS, we successfully determined the sulfite content in dried fruit samples using PSI-MS/MS.

## 2. Results and Discussion

### 2.1. Alternative Internal Standard for PSI and LC-MS/MS Analysis of Sulfite

**Figure 1:**
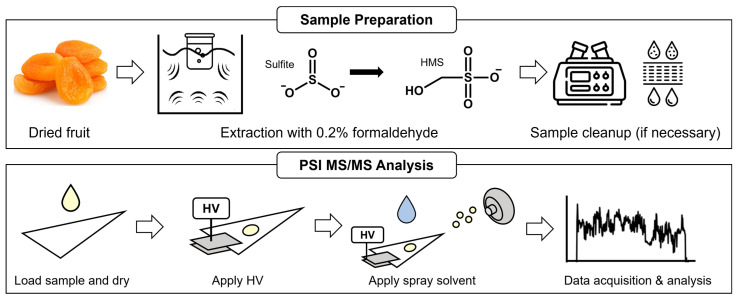
Schematic procedure of PSI-MS/MS analysis of sulfites in a dried fruit.

Schematic procedure for PSI-MS/MS analysis of sulfites in dried fruits is illustrated in Figure 1 and detailed procedure is described in Section 3. Briefly, sulfites are converted to HMS during sonication-aided extraction with 0.2% formaldehyde solution. As shown in the PSI full and tandem mass spectra of an apricot extract (Figure S1), the ion corresponding to HMS at *m*/*z* 111 and its characteristic fragment ion at *m*/*z* 81 were successfully detected using a 0.2% formaldehyde extraction solution. After centrifugation and cleanup, an IS-added sample solution is spotted onto a PSI paper tip and dried. Upon HV and spray solvent application, HMS and IS are transported to the edge of the PSI tip, sprayed, and ionized. Ion signals during spray are recorded and areas under ion chronograms are used for quantitation.

Prior to developing a quantitative method for sulfite using PSI-MS/MS, key elements of the LC-MS/MS-based method listed in the U.S. FDA Food Program Compendium of Analytical Laboratory Methods (method number: C-004.04) and related literature [1,13] were carefully reviewed. Based on the review, it became clear that for PSI-MS/MS to be a cost-effective method, it was imperative to use an effective IS that could serve as a substitute for isotopically labeled IS (Na_2_^34^SO_3_).

**Figure 2:**
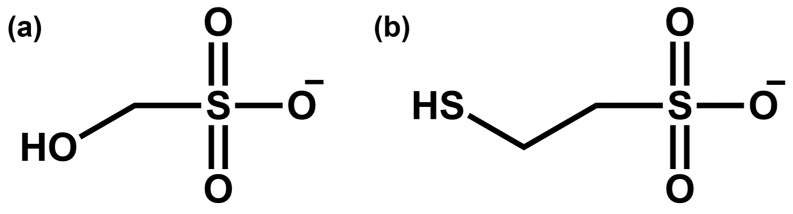
(**a**) Hydroxymethylsulfonate (HMS) and (**b**) 2-mercaptoethanesulfonate (MES).

Initially, we attempted to test chloroacetic acid, which was utilized as an IS in LC-MS/MS-based sulfite analyses before introducing Na_2_^34^SO_3_ [1]. However, we did not achieve satisfactory results with chloroacetic acid in terms of correlation coefficients for calibration curves and precision in both LC-MS/MS and PSI-MS/MS analyses. Therefore, we investigated the alkyl sulfonate salt family with similar structures to the analyte, HMS, as IS candidates and ultimately chose MES as the IS for sulfite quantitation (Figure 2). Under MS/MS, both HMS and MES produced the same fragment ion at *m*/*z* 81 (bisulfite anion). 

**Figure 3:**
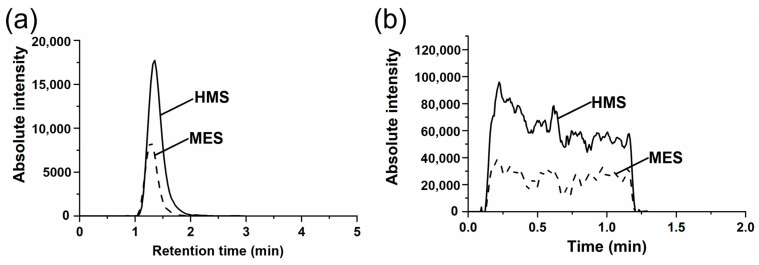
(**a**) LC-MS/MS chromatograms and (**b**) PSI-MS/MS chronograms for the transitions, *m*/*z* 111 → *m*/*z* 81 for HMS (analyte) and *m*/*z* 141 → *m*/*z* 81 for MES (IS). Data were collected from a standard solution containing (**a**) 1 ppm and (**b**) 2 ppm Na_2_SO_3_ with 0.5 ppm MES (IS).

LC-MS/MS chromatograms and PSI-MS/MS chronograms for the transitions, *m*/*z* 111 → *m*/*z* 81 for HMS (analyte) and *m*/*z* 141 → *m*/*z* 81 for MES (IS), are presented in Figure 3. For the LC-MS/MS analysis, we adopted almost identical hydrophilic interaction chromatography (HILIC) conditions to those in the previous study [1], with the only modification being a switch from the previous gradient elution mode to an isocratic elution mode. This change ensured that the IS, MES, and the analyte, HMS, eluted as closely together as possible [see Figure 3a]. In the PSI-MS/MS analysis of HMS and MES, we found that using 10 mM ammonium acetate in a 90:10 ACN/H_2_O solution, a HILIC mobile phase utilized in this study, produced stable ion signals [see Figure 3b] at more than 1 min.

For constructing calibration curves with MES as the IS, we prepared standard solutions containing 0.01 ppm–5 ppm Na_2_SO_3_ with 0.5 ppm IS and calculated the area ratios of HMS and MES. In the previous study, the linear range spanned from 0.01 to 0.8 ppm, and a calibration curve for the range of 0.01 to 4.5 ppm was created using a 1/x^2^-weighted quadratic fit [1]. We observed a similar trend in our LC-MS/MS analysis, where using MES as the IS achieved a linear response from 0.01 ppm to 1 ppm and a strong correlation coefficient (r^2^ > 0.999) employing a weighted quadratic fit for the range from 0.01 ppm to 5 ppm. This result suggests that using MES as an IS instead of Na_2_^34^SO_3_ can offer similar analytical performance. It also should be noted that MES (>98% purity) is more than 4000 times cheaper than Na_2_^34^SO_3_ (>95% purity) for an equivalent number of moles.

However, in PSI-MS/MS analysis, HMS signals began to be detected at 0.03 ppm of Na_2_SO_3_ and a quadratic calibration curve with a good correlation coefficient (r^2^ > 0.995) could be constructed for a concentration range from 0.1 ppm to 5 ppm of Na_2_SO_3_. This range, 0.1 ppm to 5 ppm of Na_2_SO_3_, corresponds to 0.05 ppm to 2.54 ppm of SO_2_, which, when converted, amounts to a range of 2.5 ppm to 127 ppm of SO_2_ in a food sample (see Section 3). Therefore, despite the poorer sensitivity and narrower dynamic range compared to LC-MS/MS, PSI-MS/MS could be useful since the regulatory threshold for SO_2_ (10 ppm) falls within this range, and most dried sulfited fruits have been shown to contain more than 20 ppm of SO_2_ [1,2]. 

### 2.2. PSI and LC-MS/MS Analysis of Sulfite with Dried Fruits

To develop and assess a PSI-MS/MS-based sulfite detection method for dried fruits, dried apricot was selected due to its typically high sulfite content [1,2,13]. Given that the primary aim of this study was to compare the PSI-MS/MS method against the conventional LC-MS/MS method, the intensive optimization of the sample preparation step was not undertaken, and we largely adhered to procedures outlined in prior studies [1,13]. Nevertheless, as the consistent generation of HMS during sonication-aided extraction is crucial, we specifically optimized the extraction time for dried apricot.

**Figure 4:**
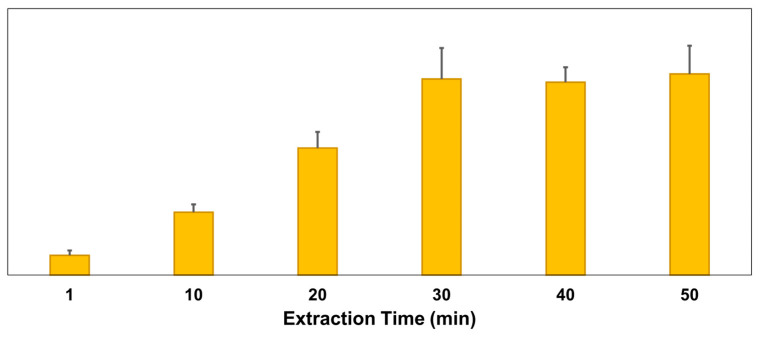
The extraction amount of sulfite, as the ratio of HMS and IS, from a dried apricot sample, varied according to the extraction time (min). Data were obtained from unfiltered extracts by using PSI MS/MS.

The amount of HMS produced as a function of extraction time for dried apricot is depicted in Figure 4. The production of HMS increased until reaching a 30 min extraction time, beyond which it remains nearly constant. Consequently, we established the extraction time at 30 min for this and other dried fruits. Importantly, the filtration process did not influence the HMS measurements by PSI-MS/MS (*p* > 0.05, *t*-test for unfiltered versus filtered after 30 min extraction). This indicates that while filtration is necessary for LC-MS/MS to prevent column clogging, it might be optional for PSI-MS/MS.

To evaluate the method’s accuracy, spike recoveries were measured in an unsulfured apricot matrix using three different concentrations (16, 32, and 80 ppm) of Na_2_SO_3_, with each concentration spike measured in triplicate. Table 1 presents the average spike recovery and percent relative standard deviation (%RSD) obtained using LC-MS/MS and PSI-MS/MS. Additionally, detailed data corresponding to Table 1 are provided in Supporting Information Table S1 and Figure S2. Although PSI-MS/MS exhibited slightly lower precision compared to LC-MS/MS, both methods achieved the target values established in the previous study [1], which are 80–120% for percent recovery and %RSD less than 16%. 

As noted in previous studies [1,13], determining an LOQ value for sulfite analysis in foods is challenging due to the presence of sulfites in formaldehyde, matrix blanks, and the IS. For dried apricots, the LOQ values were estimated to be about 0.5 ppm SO_2_ for LC-MS/MS and 2.5 ppm SO_2_ for PSI-MS/MS. Although PSI-MS/MS exhibits a five-fold higher LOQ compared to LC-MS/MS, we believe that PSI-MS/MS possesses adequate quantitative capability for evaluating the regulatory threshold of 10 µg/g SO_2_.

Finally, we prepared samples from four dried fruits (three sulfited and one unsulfured) and measured the sulfite contents in µg/g SO_2_ in triplicate using both LC-MS/MS and PSI-MS/MS on the same extracts. We chose the Black Mission variety of figs for our unsulfured fruit sample because its skin is purple-black and does not require SO_2_ treatment. A previous study has also shown that it is free from SO_2_ [25]. Summarized results are shown in Table 2, and more detailed results and representative LC-MS/MS chromatograms and PSI-MS/MS chronograms are shown in Supporting Information Table S2 and Figure S3. As shown in Table 2, both LC-MS/MS and PSI-MS/MS methods could not detect sulfite from an unsulfured fig sample and showed no significant differences (*p* > 0.05) between the methods for dried apricot and tomato samples. However, dried mango showed a significant difference between LC-MS/MS and PSI-MS/MS. Lower values observed for mango with PSI-MS/MS could be due to matrix interference, a consequence of lacking a chromatographic separation step. However, further investigation is needed to determine the exact cause for this phenomenon.

## 3. Materials and Methods

### 3.1. Materials

Sodium sulfite (Na_2_SO_3_, ≥98%) and sodium 2-mercaptoethanesulfonate (HSCH_2_CH_2_SO_3_Na, NaMES, ≥98%), formaldehyde (37%, stabilized with 10–15% methanol), ammonium acetate (98%), and ACN were purchased from Sigma-Aldrich Chemical Co. (St. Louis, MO, USA). Whatman grade 6 filter paper (0.180 mm in thickness) was obtained from Whatman International Ltd. (Maidstone, UK). Dried fruits were purchased from local grocery stores or online markets. 

### 3.2. Standard Preparation

All solutions required for the preparation of HMS standard solutions were prepared according to the procedure described in the previous LC-MS/MS study, with minor modifications [1]. Initially, a 2% formaldehyde solution in 0.05 M ammonium acetate was prepared. This was then diluted to a 0.2% formaldehyde solution using 18 MΩ water. The stock solution of Na_2_SO_3_ in 2% formaldehyde had a concentration of 10 mg/mL. To prepare working standards of 1, 10, and 100 ppm Na_2_SO_3_, the 10 mg/mL stock solution was first diluted to 1000 ppm with 18 MΩ water and then further diluted with the 0.2% formaldehyde solution, if necessary. The 25 ppm IS stock solution was prepared by dissolving NaMES in a 0.2% formaldehyde solution. On the day of analysis, this stock IS solution was further diluted with the 0.2% formaldehyde solution to achieve a final concentration of 5 ppm.

Calibration standards for both LC-MS/MS and PSI-MS/MS were prepared as follows: First, Na_2_SO_3_ at concentrations of 1, 10, or 100 ppm was diluted with the 0.2% formaldehyde solution to achieve a concentration range of 0.05–25 ppm. Then, 200 μL of the 0.05–25 ppm Na_2_SO_3_ solution was mixed with 100 μL of a 5 ppm IS solution and 700 μL of ACN, resulting in calibration standards containing 0.01–5 ppm Na_2_SO_3_ and 0.5 ppm IS.

### 3.3. Sample Preparation for Dried Fruits

We adhered to the sample preparation procedures outlined in the LC-MS/MS studies [1,13], with the exception of a few minor adjustments in sample volume and processing time. Briefly, a 1.0 g dried fruit sample was cut into small pieces (about 2 mm). Then, 9 g of 0.2% formaldehyde solution was added, and the mixture was homogenized with an ultrasonic homogenizer (VCX 130, Sonics & Materials Inc., Newtown, CT, USA) and sonicated in a sonication bath (Powersonic 405, Hwasin Tech, Daegu, Republic of Korea) for 30 min. After sonication, the homogenate was centrifuged to obtain the supernatant and the volume was adjusted to 10 mL using 0.2% formaldehyde solution. After cleanup with a C18 SPE cartridge, the extract for MS analysis was prepared by mixing 200 μL of extract with 100 μL of 5 ppm IS and 700 μL of ACN. If necessary, the extract was further diluted with 0.2% formaldehyde solution prior to mixing to ensure that the measured signal fell within the calibration range. For LC-MS/MS analysis, the supernatant was filtered using a 0.45 μm PTFE filter, although filtration was optional for PSI-MS/MS analysis. 

### 3.4. Liquid Chromatography (LC)-Tandem Mass Spectrometry (MS/MS)

HILIC separations were achieved using a Nexera X2 LC-30 AD system (Shimadzu, Kyoto, Japan) with a Syncronis™ HILIC column (50 × 2.1 mm, 5 μm, Thermo Fisher Scientific, Waltham, MA, USA). The mobile phases consisted of (A) 10 mM ammonium acetate in 90:10 ACN/H_2_O and (B) 10 mM ammonium acetate in 50:50 ACN/H_2_O. Initially, we attempted to employ a gradient elution with these mobile phases, as demonstrated in previous studies [1,13], but ultimately opted for an isocratic elution with a 90:10 (A)/(B) ratio, equivalent to 10 mM ammonium acetate in an 86:14 ACN/H_2_O mixture. The reason for choosing isocratic elution was to minimize variability during ESI by aligning the retention times of HMS and MES as closely as possible, as illustrated in the LC- MS/MS chromatogram in Figure 3. The flow rate was 400 μL/min, and the sample injection volume was 2 μL. The column oven temperature was set to 40 °C.

The LC system was interfaced with a TSQ Quantum Max triple quadrupole mass spectrometer (Thermo Scientific, Bremen, Germany) via a heated electrospray ionization (HESI) source (HESI I, Thermo) (Figure S4). The spray voltage was set to −3.0 kV, and the source heater and capillary temperatures were maintained at 50 °C and 320 °C, respectively. The sheath, auxiliary, and sweep gas pressures were adjusted to 20, 5, and 2 arbitrary units, respectively. MS/MS data were collected in the selected reaction monitoring (SRM) mode at unit mass resolution for both Q1 and Q3 (Q1 = 0.7 and Q3 = 0.7 full width half maximum). The MS/MS conditions for each monitored transition, including Q1 *m*/*z*, Q3 *m*/*z*, collision energy, and tube lens (TL) voltage, are listed in Table 3.

### 3.5. Paper Spray Ionization (PSI)-Tandem Mass Spectrometry (MS/MS)

A home-built PSI source was interfaced with a TSQ Quantum Max triple quadrupole mass spectrometer by attaching a triangular paper tip made from a Whatman grade 6 filter paper (base: 5 mm; height: 10 mm) to an HV power supply using a clip (Figure S4). For PSI-MS/MS analysis, 1 μL of sample solution was loaded onto the middle of the paper tip. After drying, a paper tip was positioned at an angle of approximately 30 degrees and placed 5 mm away from the mass spectrometer inlet. Then, a spray voltage of −3.0 kV was applied, and 15 μL of spray solution (10 mM ammonium acetate in 90:10 ACN/H_2_O) was administered onto the paper tip. Subsequently, MS/MS data were collected in the SRM mode, with conditions identical to those specified for the LC-MS/MS experiments (see Table 3).

### 3.6. Quantitation

Calibration curves were constructed by plotting the area ratio of HMS (analyte) and MES (internal standard) against the Na_2_SO_3_ concentration. For calculating the area ratio values, the chromatographic peak area was utilized for LC-MS/MS, whereas the area under ion chromatogram was used for PSI-MS/MS. Both area values were measured using Xcalibur data processing software (Version 2.2, Thermo Fisher Scientific). Concentration values determined by interpolation were adjusted for the dilution factor. In the case of food samples, all concentration values were reported as μg of SO_2_ per g of food material, employing the molar mass ratio of SO_2_ (64 g/mol) to Na_2_SO_3_ (126 g/mol).

## 4. Conclusions

In this communication, we demonstrated initial results of sulfite quantitation using the PSI-MS/MS method, compared to the conventional LC-MS/MS approach. The results indicate that the new internal standard (IS), MES, provided analytical performance comparable to conventional isotopically labeled IS. Although PSI-MS/MS exhibited slightly lower sensitivity than LC-MS/MS, it still remains effective for measuring sulfites in dried fruits for regulatory purposes. Moving forward, we aim to optimize the PSI-MS/MS method to enhance its sensitivity and perform the full validation of the optimized method across various food matrices.

## Figures and Tables

**Table 1:** Average percent recovery and percent relative standard deviation (*n =* 3) for three concentration spikes within the dried apricot matrix measured using the LC-MS/MS and PSI-MS/MS.

Method	16 ppm Na_2_SO_3_	32 ppm Na_2_SO_3_	80 ppm Na_2_SO_3_
LC-MS/MS	91 ± 3	99 ± 4	106 ± 4
PSI-MS/MS	88 ± 9	96 ± 10	109 ± 8

**Table 2:** Sulfite contents (in µg/g SO_2_) in four dried fruits determined by LC-MS/MS and PSI-MS/MS methods.

Method	Apricot	Mango	Tomato	Fig ^1^
LC-MS/MS	463 ± 27	55 ± 3	224 ± 8	ND ^2^
PSI-MS/MS	482 ± 44	42 ± 6	213 ± 6	ND

^1^ Dried fig we analyzed is sold as unsulfured. ^2^ ND denotes not detected.

**Table 3:** MS/MS conditions for monitored transitions.

Compound ID	Q1 *m*/*z*	Q3 *m*/*z*	CE (V)	TL (V)
HMS	111	81	27	25
HMS	111	80	14	25
MES (IS)	141	81	17	42
MES (IS)	141	80	31	42

## Data Availability

The data presented in this study are available on request from the corresponding author.

## Supplementary Materials

The following supporting information can be downloaded at https://www.mdpi.com/article/10.3390/molecules29102192/s1. Figure S1. PSI mass spectra of dried apricot extracts; Figure S2. Calibration curves for sulfite quantitation; Figure S3. Representative LC-MS/MS chromatograms and PSI-MS/MS chronograms of dried fruit extracts; Figure S4. Analytical platforms used in this study. Table S1. Detailed results for Table 1; Table S2. Detailed results for Table 2.
