# Supplementary material for: Determination of Sulfites in Dried Fruits by Paper Spray Ionization Tandem Mass Spectrometry
Source: Molecules. 2024 May 8;29(10):2192. doi: 10.3390/molecules29102192 (PMC11124160; doi:10.3390/molecules29102192)
Supplement: Supplementary file 1 [file molecules-29-02192-s001.zip › molecules-2984195-supplementary.pdf]

Supporting Information

Determination of sulfites in dried fruits by paper spray ionization tandem mass spectrometry

Donghoon Lee [1], Heejin Ro [1], Seoyoung Hwang [1], Minkyu Lee [1], Hyebeen Kim [1], Jaeyoung Heo [1], and Sangwon Cha [1,*]

[1]Department of Chemistry, Dongguk University, Seoul 04620, Republic of Korea; chasw@dongguk.edu

*Correspondence: chasw@dongguk.edu; Tel.: +82-2-2260-8907 (S.C.)

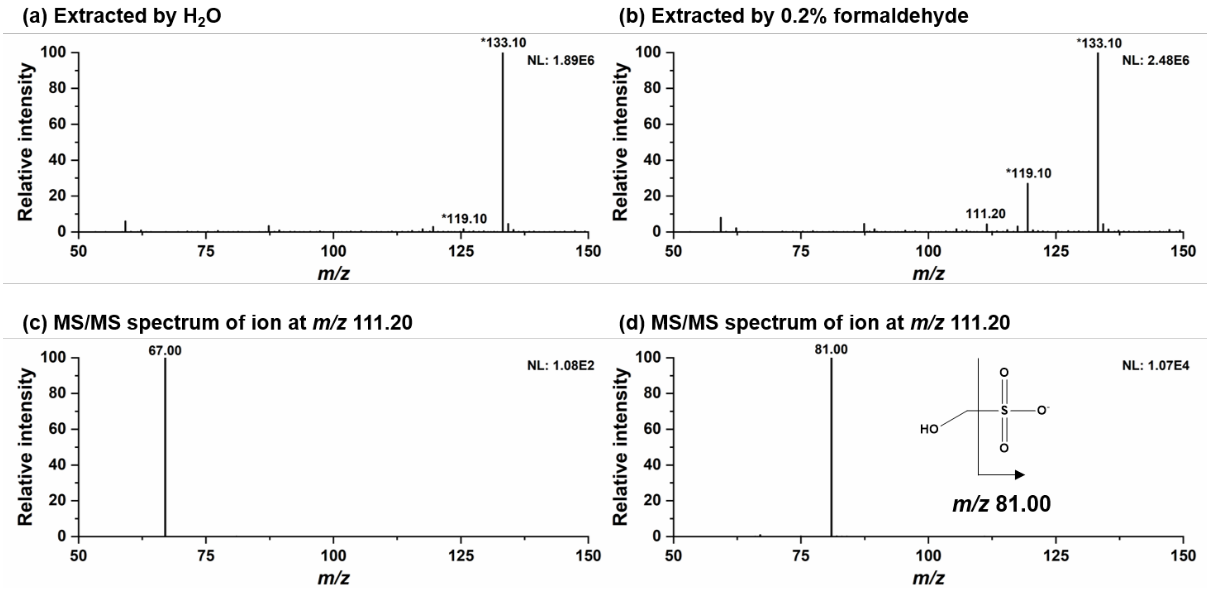

Figure S1. (a and b) PSI mass spectra of dried apricot extracts. Extraction was performed with (a) H_2O as a control and (b) 0.2% formaldehyde solution. It should be noted that an ion signal from hydroxymethyl sulfonate (HMS) was detected at *m/z* 111.20 with 0.2% formaldehyde solution (b), whereas any signal from HMS was not detected in the control (a). (c and d) Product ion spectra (MS/MS spectra) for the ion at *m/z* 111.20 from dried apricot extracts using (c) H_2O and (d) 0.2% formaldehyde solution. It should be also noted that a characteristic fragment ion at *m/z* 81.00 was detected only from a sample extracted with 0.2% formaldehyde (d). Full mass spectra and MS/MS spectra were collected using a linear ion trap mass spectrometer (Thermo Finnigan LTQ XL, Mountain View, CA, USA). NL denotes normalized level.

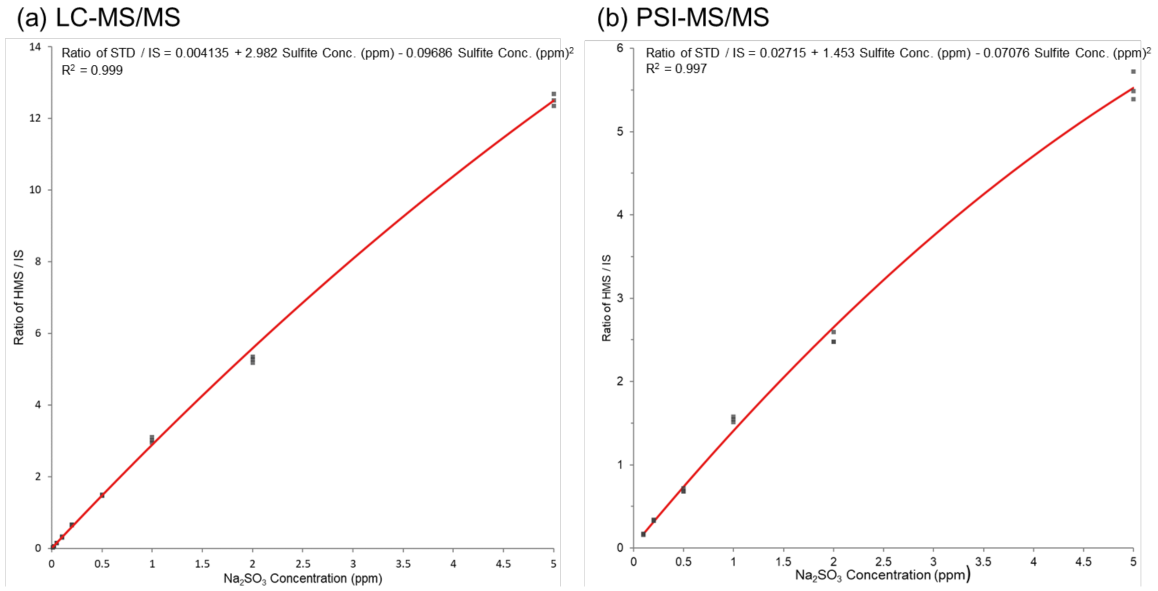

Figure S2. Calibration curves constructed for investigating average percent recovery for three concentration spikes using (a)LC-MS/MS and (b)PSI-MS/MS. $1/x^2$-weighted quadratic fit was performed using Analyse-it software (Analyse-it Software Ltd, Leeds, UK).

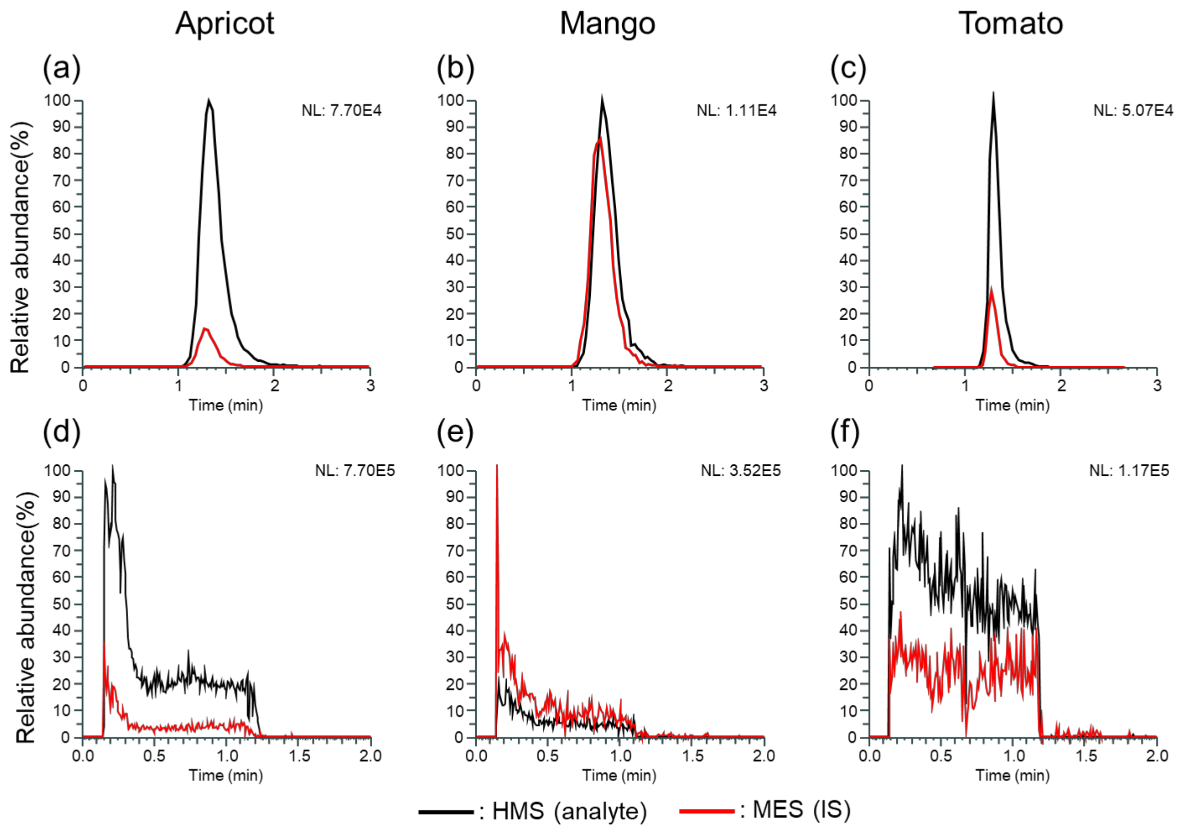

Figure S3. Representative (a – c) LC-MS/MS chromatograms and (d – f) PSI-MS/MS chronograms of dried fruit extracts. Dried fruit samples analyzed were (a and d) apricot, (b and e) mango, and (c and f) tomato samples. Black traces were obtained from the transition *m/z* 111 → *m/z* 81 (HMS, analyte) and red traces were obtained from the transition *m/z* 141 → *m/z* 81 (MES, IS). NL denotes normalized level.

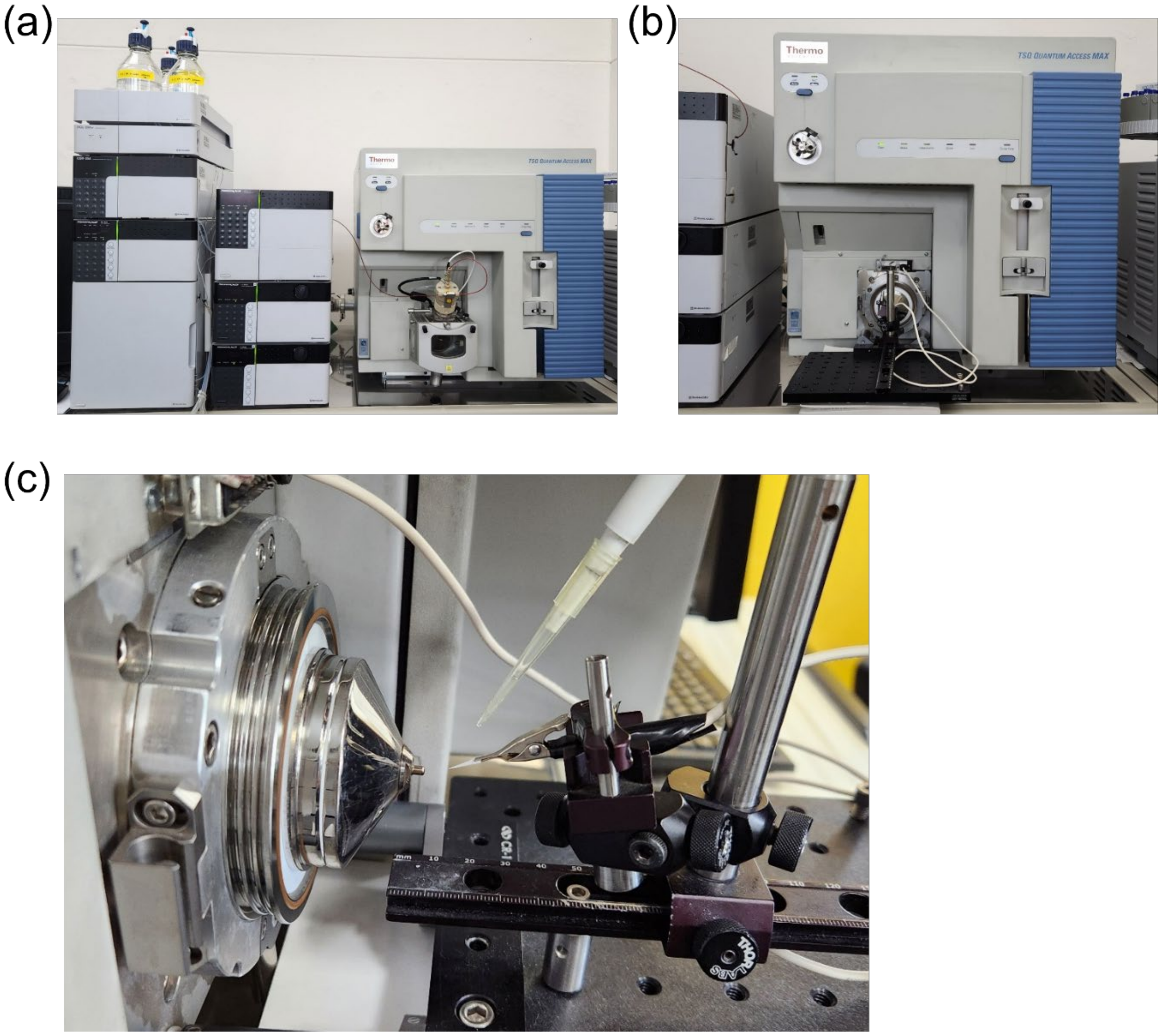

Figure S4. Analytical platforms used in this study, (a) LC-MS/MS and (b and c) PSI-MS/MS.

Table S1. Detailed results for average percent recovery and percent relative standard deviation (n=3) for three concentration spikes within the dried apricot matrix measured using the LC-MS/MS and PSI-MS/MS.

**Na_2SO_3 spike concentration (ppm)**	**Measured HMS/IS ratio**	**Concentration determined from the calibration curve[1)] (ppm)**	**Converted concentration with dilution factor (ppm)**	**Percent recovery (%)**
**LC-MS/MS**				
16	0.895	0.302	15.09	94.33
	0.841	0.283	14.15	88.47
	0.845	0.284	14.22	88.89
			Average ± Std. dev.	**90.56 ± 3.27**
32	1.795	0.613	30.64	95.77
	1.815	0.620	30.99	96.86
	1.932	0.661	33.04	103.26
			Average ± Std. dev.	**98.63 ± 4.05**
80	4.584	1.621	81.05	101.32
	4.786	1.697	54.85	106.07
	4.933	1.753	87.64	109.54
			Average ± Std. dev.	**105.64 ± 4.13**

**Na_2SO_3 spike concentration (ppm)**	**Measured HMS/IS ratio**	**Concentration determined from the calibration curve[2)] (ppm)**	**Converted concentration with dilution factor (ppm)**	**Percent recovery (%)**
**PSI-MS/MS**				
16	0.476	0.313	15.67	97.94
	0.429	0.280	14.01	87.57
	0.391	0.253	12.66	79.14
			Average ± Std. dev.	**88.21 ± 9.42**
32	0.834	0.571	28.57	89.28
	0.854	0.586	29.30	91.56
	0.995	0.689	34.45	107.67
			Average ± Std. dev.	**96.17 ± 10.02**
80	2.175	1.603	80.17	100.21
	2.374	1.767	88.36	110.45
	2.468	1.845	92.27	115.34
			Average ± Std. dev.	**108.67 ± 7.72**

1) Ratio of HMS / IS = 0.004135 + 2.982 Sulfite Conc. (ppm) - 0.09686 Sulfite Conc. $(ppm)^2$ (R^2 = 0.999)

2) Ratio of HMS / IS = 0.02715 + 1.453 Sulfite Conc. (ppm) - 0.07076 Sulfite Conc. $(ppm)^2$ (R^2 = 0.997)

Table S2. Detailed results for sulfite contents (in μg/g SO_2) in dried fruits determined by LC-MS/MS and PSI-MS/MS methods.

**Dried fruit**	**Measured HMS/IS ratio**	**Concentration determined from the calibration curve[1)] (ppm)**	**SO_2 concentration (μg/g)[3)]**
**LC-MS/MS**			
Apricot	7.713	3.402	432.05
	8.490	3.798	482.24
	8.388	3.745	475.54
		Average ± Std. dev.:	**463.27 ± 27.25**
Mango	1.126	0.437	55.44
	1.178	0.458	58.14
	1.069	0.414	52.51
		Average ± Std. dev.:	**55.36 ± 2.82**
Tomato	4.083	1.695	215.24
	4.247	1.768	224.48
	4.370	1.823	231.45
		Average ± Std. dev.:	**223.72 ± 8.13**

**Dried fruit**	**Measured HMS/IS ratio**	**Concentration determined from the calibration curve[2)] (ppm)**	**SO_2 concentration (μg/g)**
**PSI-MS/MS**			
Apricot	5.004	3.745	475.56
	5.422	4.162	528.54
	4.719	3.475	441.23
		Average ± Std. dev.:	**481.78 ± 43.99**
Mango	0.542	0.311	39.5
	0.527	0.301	38.24
	0.666	0.390	49.54
		Average ± Std. dev.:	**42.43 ± 6.19**
Tomato	2.522	1.668	211.78
	2.487	1.641	208.42
	2.606	1.730	219.64
		Average ± Std. dev.:	**213.28 ± 5.76**

1) Ratio of HMS / IS = 0.0433 + 2.512 Sulfite Conc. (ppm) - 0.07578 Sulfite Conc. (ppm)^2 (R^2 = 0.999)

2) Ratio of HMS / IS = 0.04839 + 1.612 Sulfite Conc. (ppm) - 0.07709 Sulfite Conc. (ppm)^2 (R^2 = 0.997)

3) $SO_2\ conc.\,(\mu g/g) =$
$Na_2SO_3\ conc.\,(\mu g/mL) \times \frac{Extract\ volume\ (10\ mL)}{Fruit\ sample\ amount\ (1.0\ g)} \times \left(\frac{1000\ \mu L\ sample\ solution}{200\ \mu L\ extract}\right) \times df\ (5)\ \times \left(\frac{64\ g/mol\ SO_2}{126\ g/mol\ Na_2SO_3}\right)$

- Na_2SO_3 conc.: determined concentration from the calibration curve
- $\left(\frac{1000\ \mu L\ sample\ solution}{200\ \mu L\ extract}\right)$: 200 μL of extract was mixed with 100 μL of IS and 700 μL of ACN for MS analysis.
- *df*: dilution factor if extract was further diluted in order for the HMS/IS ratio to fall within a calibration range. Since dried fruit extract was further diluted five times, *df* of 5 was applied in this case.
- $\left(\frac{64\ g/mol\ SO_2}{126\ g/mol\ Na_2SO_3}\right)$: conversion term from Na_2SO_3 concentration to SO_2 concentration
